# Amyloid *β* Peptide-Induced Changes in Prefrontal Cortex Activity and Its Response to Hippocampal Input

**DOI:** 10.1155/2017/7386809

**Published:** 2017-01-03

**Authors:** Ernesto Flores-Martínez, Fernando Peña-Ortega

**Affiliations:** Departamento de Neurobiología del Desarrollo y Neurofisiología, Instituto de Neurobiología, Universidad Nacional Autónoma de México, 76230 Querétaro, QRO, Mexico

## Abstract

Alterations in prefrontal cortex (PFC) function and abnormalities in its interactions with other brain areas (i.e., the hippocampus) have been related to Alzheimer Disease (AD). Considering that these malfunctions correlate with the increase in the brain's amyloid beta (A*β*) peptide production, here we looked for a causal relationship between these pathognomonic signs of AD. Thus, we tested whether or not A*β* affects the activity of the PFC network and the activation of this cortex by hippocampal input stimulation* in vitro*. We found that A*β* application to brain slices inhibits PFC spontaneous network activity as well as PFC activation, both at the population and at the single-cell level, when the hippocampal input is stimulated. Our data suggest that A*β* can contribute to AD by disrupting PFC activity and its long-range interactions throughout the brain.

## 1. Introduction

The prefrontal cortex (PFC) is implicated in cognitive processes including working memory, temporal processing, decision making, flexibility, and goal-oriented behavior [1–4]. Alterations in some of these processes are observed in Alzheimer's Disease (AD) patients [5, 6], and they correlate with amyloid beta (A*β*) peptide accumulation in the PFC and other related brain areas [7, 8]. Similar alterations in PFC function are observed in AD transgenic mice [9–11], which also correlate with increased A*β* levels in the PFC and other connected brain areas [9, 11]. These findings suggest that A*β* can alter PFC functionality [11]. In fact, alterations in PFC synaptic transmission [12] and plasticity [11], as well as in cell excitability [13] and in network activity [14], have been reported in AD transgenic mice. Some data indicate that these deleterious effects might be produced directly by the presence of A*β* in the PFC [15–17].

Alterations in PFC-controlled behaviors [18–20] and function [18, 19] can also be induced by intrahippocampal application of A*β*, which indicates that altered PFC function can also be induced by dysfunctional connectivity with other brain areas [18–20]. The hippocampal connection with the PFC consists of excitatory glutamatergic fibers that synapse on both PFC pyramidal neurons [21–23] and interneurons [23–25]. This connection allows the synchronization between these two structures, in different frequency patterns, which correlates with animals' behavioral performance in PFC functions mentioned above [18, 19, 26–28]. Moreover, AD patients exhibit alterations in PFC coupling with the hippocampus and in the functions that rely on this reciprocal connection [29–32]. The possibility that alterations in the synaptic interactions between the hippocampus and the PFC also contribute to A*β*-induced pathology prompted us to test the effects of A*β* on the PFC activity isolated in a brain slice [23] as well as on the PFC activation induced by the stimulation of the hippocampal fibers preserved in a PFC slice preparation developed by Parent et al. [23]. We found that A*β* inhibits both PFC spontaneous network activity and PFC activation, both at the population and at the single-cell level, induced by hippocampal fiber-activation. Our data suggest that A*β* contributes to PFC dysfunction by a direct effect on its network activity as well as by a reduction in its synaptic innervation from the hippocampus.

## 2. Materials and Methods

### 2.1. Ethics Statement

Approval of the Bioethics Committee of the Instituto de Neurobiología at Universidad Nacional Autónoma de México was granted for all the experimental procedures (protocol number 91.A), which were carried out according to the guidelines of the Institutional Animal Care and Use Committee Guidebook (NIH publication 80-23, Bethesda, MD, USA, 1996).

### 2.2. Subjects

Specific pathogen-free Wistar rats (8–12 weeks old) were obtained from our breeding colony located in the facility of the Instituto de Neurobiología. All animals were housed in groups of four animals, in transparent acrylic cages located in ventilated racks (12 to 15 complete air changes per hour) at constant temperature (21 ± 1°C) and humidity (50 ± 10%) and maintained on a 12-h/12-h light/dark cycle with free access to food (Irradiated Picolab Rodent Diet 20, PMI) and water* ad libitum*.

### 2.3. Amyloid Beta Preparation

A*β*_42_ peptide was obtained from BACHEM (Heidelberg, Germany). The oligomerization procedure was performed as previously described [33, 34]. Briefly, solid A*β*_1-42_ peptide was dissolved in 1,1,1,3,3,3-hexafluoro-2-propanol (HFIP) to a final concentration of 1 mM. This solution was incubated for 60 min at room temperature, the HFIP was evaporated overnight, and DMSO was added to prepare a 5 mM solution. Then, by adding F12 medium (MF12), a new solution of A*β*_42_ was obtained with a final concentration of 100 *μ*M (100 pmoles/*μ*L). This solution was incubated for 24 h at 5°C and then centrifuged at 14,000 ×g at 4°C for 10 min. A*β* oligomers found in the supernatant were collected and maintained at 4°C until being used for experiments. Previous characterization of our solution indicates that it contains a mixture of A*β* aggregates, with hexamers as the main A*β* oligomeric form present [34].

### 2.4. Prefrontal Cortex Slice Preparation

Animals were anesthetized with sodium pentobarbital (62 mg/Kg) and perfused transcardially with cold modified artificial cerebrospinal fluid containing (in mM) 238 sucrose, 3 KCl, 2.5 MgCl_2_, 25 NaHCO_3_, and 30 D-glucose, pH 7.4, and bubbled with carbogen (95% O_2_ and 5% CO_2_). Then, the brain was removed and dissected in ice-cold artificial cerebrospinal fluid (aCSF) containing (in mM) 119 NaCl, 3 KCl, 1.5 CaCl_2_, 1 MgCl_2_, 25 NaHCO_3_, and 30 D-glucose, pH 7.4, and bubbled with carbogen. The cerebellum was removed, both hemispheres were mounted onto an agar block with a 10–12° inclination [23], and coronal slices containing both the PFC (400 *µ*m thick) and the hippocampal bundle were obtained using a vibratome (Microm HM 650 V, Thermo Scientific, USA). Only one slice was obtained per animal. The slices were left to recover at room temperature for at least 60 min before any further experimental manipulation.

### 2.5. Electrophysiological Recordings

For extracellular field population recordings, PFC slices were transferred to a submerged recording chamber continuously perfused at 15–17 ml/min with oxygenated aCSF maintained at 30–32°C. The field recordings were obtained using borosilicate electrodes (0.5–1 MΩ) filled with aCSF and positioned on layer 5-6 of the prelimbic region of the PFC [23]. PFC spontaneous activity was recorded for 20 min to obtain the basal network activity. Thereafter, A*β* was added to the bath, and its effects were recorded for 1 h. Finally, 1 mM lidocaine was added to the bath to block neural activity, as a control for the viability of the slice [35]. Alternatively, the hippocampal axonal bundle was stimulated electrically with a concentric bipolar microelectrode (FHC Inc., Bowdoin, ME, USA) [36–38]. The synaptic potentials were evoked by trains of 5 pulses at different frequencies (5, 10, 20, and 50 Hz). Each stimulus in the train had a duration of 100-*μ*s and was delivered at 0.04 Hz. The stimulus intensity was adjusted in each experiment and for each preparation to evoke a response of 50% maximal amplitude [36–38]. After recording control potentials, 30 nM A*β* was added to the bath, and its effects on the synaptic transmission were monitored for 60 min. Then 10 *µ*M APV and 10 *µ*M CNQX were added to the bath to block all glutamatergic transmission. Finally, 1 mM lidocaine was added to the bath to block any neuronal activity.

### 2.6. Calcium Imaging

PFC slices were incubated at room temperature, in the dark, for 2 h in the presence of 10 *µ*M Fluo-8 AM (Invitrogen) and 0.3% pluronic acid in aCSF equilibrated with carbogen [37, 39–41]. Then, after a recovery period of 2 h, the slices were transferred and immobilized, with a nylon mesh, into a perfusion chamber on a microscope adapted to an epifluorescence system (Eclipse E600FN; Nikon, Melville, NY). Slices were continuously perfused with aCSF equilibrated with carbogen at 30–32°C. Excitation at 488 nm was performed with a Lambda LS illuminator (Sutter Instrument, Novato, CA), and images were acquired with a cooled digital camera (CoolSNAP-ES, Roper Scientific, Tucson, AZ). The imaging software used was RS Image (Photometrics; Roper Scientific, Tucson, AZ), and the imaged field was 800 × 600 *µ*m. Short movies (175 s, 40-*µ*s exposure, four images per second) were taken. Cells active during the experiment were analyzed. The hippocampal axonal bundle was stimulated electrically as described above in control conditions and in the presence of A*β*.

### 2.7. Data Analysis

For all electrophysiological experiments, the signal was amplified and filtered (highpass, 0.5 Hz; lowpass, 1.5 KHz) with a wide-band AC amplifier (Grass Instruments, Quincy, MA, USA). All recordings were digitized at 9 KHz and stored on a personal computer with an acquisition system from National Instruments (Austin, TX, USA) using custom-made software designed for the LabView environment. The recordings obtained were analyzed off-line. All evoked synaptic responses were measured from the start of the stimulation artifact to the valley of the synaptic response in Clampfit (Molecular Devices). Three 10-sec segments of each condition were analyzed using a Fast Fourier Transform Algorithm with a Hamming window also in Clampfit. The power spectra of all segments were averaged and normalized to the basal spontaneous activity of each individual experiment.

For calcium imaging, image processing was carried out with ImageJ (v.1.36, National Institutes of Health) and custom-made programs in LabView and MATLAB [40, 41]. All active neurons in a field were semiautomatically identified, and their mean fluorescence was measured as a function of time. Single pixel noise was discarded using a 5-pixel ratio mean filter. Calcium-dependent fluorescence signals were computed as (Fi − Fo)/Fo, where Fi is the fluorescence intensity at any frame and Fo is the resting fluorescence, that is, average fluorescence of the first four frames of the movie. Calcium signals were detected based on a threshold value given by their first time derivative (2.5 times the standard deviation (SD) of the noise value). Thus, we obtained a *C* × *F* binary matrix, where *C* represents the number of active cells and *F* the number of frames for each movie. Recordings were inspected manually to remove artifacts and slow calcium transients which are likely to correspond to glial cells [37, 40]. After defining all neuronal-like calcium transients, we built raster plots and quantified both the number of active neurons per bin (250 ms) and the number of neuronal-like calcium transients per neuron (cell-activation instances).

All data are expressed as mean ± standard error of the mean (SEM). In most cases the data distribution was markedly skewed, and hence we used a Mann–Whitney Rank Sum Test or a Kruskal-Wallis One-Way Analysis of Variance on Ranks followed by Dunn's Method for multiple comparisons. Differences with statistical significance are denoted by *p* < 0.05.

## 3. Results

**Figure 1:**
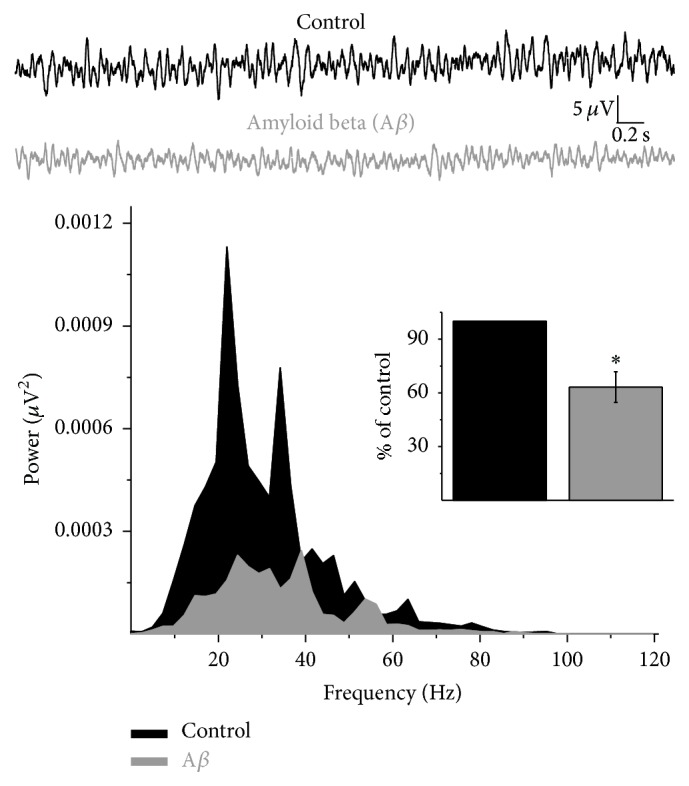
A*β* inhibits PFC spontaneous population activity. At the top, representative traces of PFC spontaneous activity are shown recorded in basal conditions (black traces) and after 60 min of continuous application of A*β* (30 nM; gray traces). Their corresponding power spectra are shown at the bottom, and the integrated power for each condition (basal power set as 100%) is shown as an inset. Note that A*β* application significantly reduces the power of the PFC spontaneous population activity. Data are presented as mean ± SEM. ^*∗*^ *p* < 0.05 versus control activity (*n* = 10 slices).

To evaluate the effect of A*β* on the general activity of the prelimbic region of the PFC, we measured its spontaneous population activity* in vitro* (Figure 1). Spontaneous prefrontal network recordings in slices showed low-voltage neuronal activity that includes a broad range of frequency components (Figure 1; *n* = 10; meaning 10 slices obtained from 10 animals, with only one slice per animal). As previously shown for other neuronal networks [34–37], this activity is reduced by the application of 30 nM A*β* (Figure 1, representative traces and power spectra). Analysis of the integrated power (from 1 to 120 Hz) showed a significant reduction of the prefrontal spontaneous network activity 60 min after A*β* application (to 63.2 ± 8.5% of basal activity, *p* < 0.05; *n* = 10) (Figure 1, inset bar graph).

**Figure 2:**
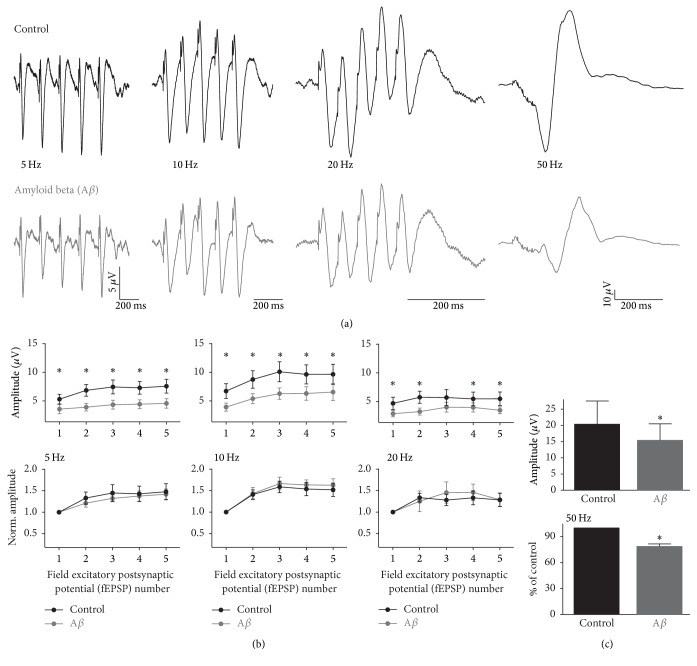
A*β* inhibits hippocampal input into PFC. (a) Representative traces of EPSPs recorded in the PFC and evoked by stimulation of the hippocampal bundle at different frequencies (5–50 Hz) are shown. The EPSPs are represented both under basal conditions (black traces) and after 60 min of continuous application of A*β* (30 nM; gray traces). (b) The amplitude of each EPSP during the different trains is plotted as the absolute value and also as the value normalized to the first EPSP (EPSP_n_/EPSP_1_ = Norm. Amplitude). The mean amplitudes of the EPSPs are represented under basal conditions (black dots/lines) and after 60 min of continuous application of A*β* (30 nM; gray dots/lines). (c) The amplitudes of the “compound” EPSPs are represented in the bar graphs quantified as absolute values (upper graph) and also after normalizing to the control (set at 100%; lower graph). Note that A*β* application significantly reduces the synaptic input from the hippocampal fibers into the PFC. Data are presented as mean ± SEM. ^*∗*^ *p* < 0.05 versus control activity (*n* = 10 slices).

To evaluate the effect of A*β* on the hippocampal input into the PFC, we initially measured the field excitatory postsynaptic potentials (fEPSPs) in the PFC induced by the stimulation of the hippocampal input at different frequencies [42]. The repetitive stimulation of the hippocampal fibers induces fEPSPs in the PFC that exhibit different degrees of facilitation depending of the stimulation frequency [42] (Figure 2(a)). For instance, the amplitude of the fifth fEPSP increases to 141.9 ± 12.0% of the first fEPSP when the stimulation is applied at 5 Hz (Figure 2(b)). When the stimulation is applied at 10 Hz, the amplitude of the fifth fEPSP increases to 162.7 ± 14.8% of the first fEPSP (Figure 2(b)). When the stimulation is applied at 20 Hz, the amplitude of the fifth fEPSP increases only to 128.7 ± 15.0% of the first fEPSP (Figure 2(b)). When the stimulation was applied at 50 Hz the individual fEPSPs get mixed into a “compound” fEPSP that does not allow individual fEPSPs to be evaluated accurately. Thus, in this case, we quantified the maximal amplitude of the compound fEPSP (20.3 ± 6.8 *µ*V; (Figure 2(c)). Bath application of A*β* reduces the amplitude of the fEPSPs, as well as that of the compound fEPSP (Figure 2(a)), regardless of the stimulation frequency or the fEPSP number (1 to 5; Figure 2(b); *p* < 0.05), except for the third fEPSP of the stimulation applied at 20 Hz, for which no significant reduction was observed after A*β* application (81.1 ± 13.2% of control, Figure 2(c); *p* = 0.07). In spite of this generalized reduction in synaptic coupling produced by bath application of A*β*, no change in the synaptic facilitation was observed for any of the fEPSP trains evoked at 5, 10, or 20 Hz (Figure 2(b); *p* < 0.05). This can be seen more clearly when the amplitude of each fEPSP in the train is normalized to the amplitude of the first fEPSP (set as 1; Figure 2(b)). In the case of the compound fEPSP, A*β* application significantly reduced the maximal amplitude to 78.6 ± 2.8% of control (Figure 2(c); *p* < 0.05). Thus, these results indicate that bath application of A*β* produces a generalized reduction in the synaptic neurotransmission provided by the hippocampus into the PFC.

**Figure 3:**
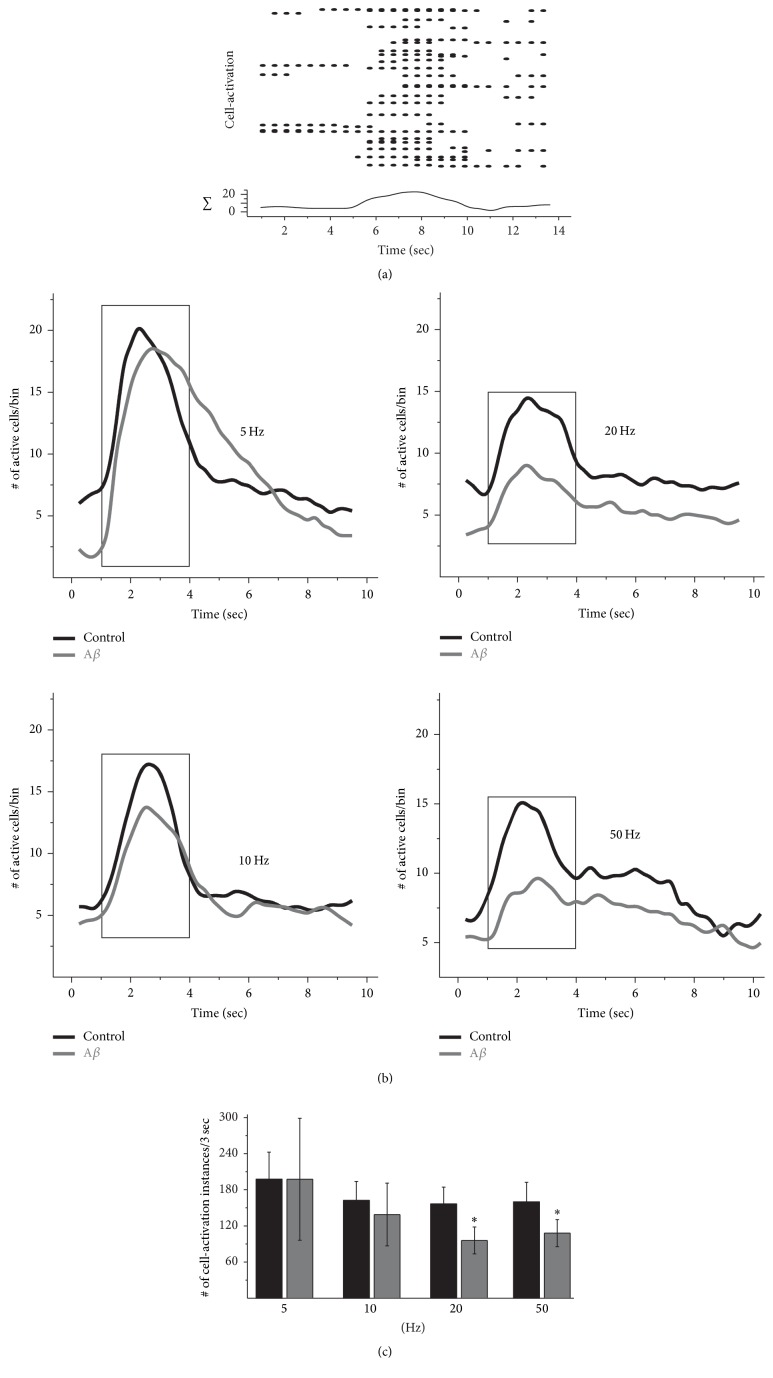
A*β* inhibits PFC-neuron recruitment by hippocampal input stimulation. (a) A representative raster plot shows the activity of PFC neurons and its response to the stimulation of the hippocampal bundle (delivered at second five). Each row represents the activity of a single cell, and each dot represents a cell-activation (i.e., calcium transient). At the bottom, the sum of all the cell-activation instances per bin (bin = 250 ms) is quantified. (b) The graphs represent the mean sums of cell-activation instances per bin for all the frequencies tested. The mean sums are represented both under basal conditions (black lines) and after 60 min of continuous application of A*β* (30 nM; gray lines). (c) Quantification of the number of cell-activation instances in 3 sec (represented by the rectangle) after the different stimulation trains is presented. Note that A*β* application significantly reduces PFC-neuron recruitment when the hippocampal bundle stimulation is applied at high frequencies. Data are presented as mean ± SEM. ^*∗*^ *p* < 0.05 versus control activity (*n* = 5 slices).

To evaluate the effect of A*β* on the hippocampal input into the PFC at the cellular level, we measured the calcium transients induced in single neurons by the stimulation of the hippocampal input. First, we found that the stimulation of the hippocampal fibers recruits PFC neurons, increasing their calcium transients (cell-activation instances) for several seconds (Figure 3). Then, we observed that there is a differential recruitment of PFC neurons depending on the stimulation frequency [42] (Figure 3). In control conditions, a maximal number of PFC neurons are recruited when hippocampal fibers are stimulated at 5 Hz (21.2 ± 5.4 neurons; *n* = 5 slices; Figure 3(b)). This cell recruitment is significantly reduced when stimulation is applied both at 10 Hz (17.4 ± 5.44 neurons; *p* < 0.05) and at 20 Hz (15.4 ± 3.7 neurons; *p* < 0.05), whereas it tends to be reduced when stimulation is applied at 50 Hz (15.7 ± 2.8 neurons; *p* < 0.09). The maximal number of PFC neurons recruited after hippocampal stimulation is reduced in the presence of A*β*, compared to control conditions, when the hippocampal fibers are stimulated at 20 Hz (to 66.4 ± 13.7% of control; *p* < 0.05; Figure 3(b) right upper part) and 50 Hz (to 71.0 ± 13.2% of control; *p* < 0.05; Figure 3(b) right lower part). No significant differences versus control conditions were observed in the maximal number of PFC neurons recruited after hippocampal stimulation in the presence of A*β* when the stimulation was applied at 5 and 10 Hz (Figure 3(b) left upper and lower part). The result was similar when the total number of cell-activation instances in 3 seconds was quantified (Figure 3(c)). Compared to control conditions, the total number of cell-activation instances was significantly reduced in the presence of A*β* when the hippocampal fibers were stimulated at 20 Hz (to 59.2 ± 8.4% of control; *p* < 0.05; Figure 3(c)) and 50 Hz (to 68.6 ± 11.1% of control; *p* < 0.05; Figure 3(c)), but there were no significant differences when the hippocampal fiber stimulation was applied at 5 and 10 Hz (Figure 3(c)).

## 4. Discussion

Here, we found that A*β* inhibits PFC spontaneous network activity as well as the PFC activation induced by hippocampal fiber-activation both at the population and at the single-cell level, suggesting that A*β* might contribute to PFC dysfunction by a direct effect on this network as well as by a reduction in its synaptic innervation. This finding might constitute the cellular basis of several cognitive deficits that can be produced by PFC dysfunction and/or disrupted PFC-hippocampal coupling and are observed in both AD patients and AD transgenic models.

Our finding that A*β* inhibits PFC spontaneous network activity is very similar to observations by our group and others that direct application of A*β* inhibits spontaneous network activity in a variety of networks including the olfactory bulb [43], the entorhinal cortex [44], and the hippocampus [37, 45]. In fact, a previous finding already indicated that direct application of A*β* inhibits synchronized activity induced by calcium depletion in PFC slices [46]. In this case, inhibition of A*β*-induced network activity was related to changes in cell excitability [46]. This A*β*-induced inhibition of cell excitability was found to be more prominent in PFC interneurons [17]. This finding correlates with those obtained previously in our laboratory, which show that, despite the lack of effect of A*β* on action potential firing in hippocampal pyramidal cells, the presence of A*β* does induce a reduction in subthreshold membrane potential oscillation [37]. This latter effect might contribute to the A*β*-induced action potential desynchronization in the hippocampus that contributes to the inhibition of its neural network activity [45]. Aside from the changes in cell excitability, the inhibition of neural network activity induced by A*β* has also been related to a reduction in both excitatory [34, 37, 47, 48] and inhibitory [47, 48] synaptic transmission. In fact, these findings are consistent with the observation that A*β* reduced cholinergic modulation of the inhibitory transmission in the PFC [17]. Altogether, the changes in cell excitability and synaptic transmission might contribute to the A*β*-induced inhibition of PFC network activity [15–17]. It is important to point out that our finding that A*β* inhibits PFC spontaneous network activity coincides with studies showing changes in PFC network function in AD animal models [14, 18, 19] and AD patients [29–31, 49], suggesting that this pathological process can contribute to PFC dysfunction in AD.

AD-associated PFC dysfunction also seems to be the result of reduced PFC coupling to other brain areas [18–20, 29–31, 49]. One PFC connection that is disturbed in AD is the PFC-hippocampal coupling [29–31]. As was already mentioned, alterations in PFC-controlled behaviors [18–20] and function [18–20] can be induced by intrahippocampal injection of A*β*. It is well known that A*β* affects hippocampal function both* in vivo* [37, 50] and* in vitro* [33, 45, 51] and, here, we show that A*β* can affect hippocampal input into the PFC. As this connection is required for the proper synchronization between these two structures and for normal PFC function [18, 19, 26, 27, 32], it is likely that PFC-hippocampal uncoupling could contribute to A*β*-induced pathology and, perhaps, to AD. Considering that PFC-hippocampal coupling occurs at a variety of different oscillatory frequencies [32, 52, 53], we tested whether A*β* affects hippocampal input when it is stimulated at different frequencies. Whereas we observed a generalized reduction in PFC activation at all frequencies tested, the inhibition was more prominent, at least at the unicellular level, when the stimulation was delivered at high frequencies (Figure 3). One possible explanation is that this connection is tuned to synchronize the two circuits at low frequencies [26, 35, 42] and, thus, not only is the hippocampal input more efficient in recruiting the PFC at low frequencies [26, 35, 42] but it also renders the connection less vulnerable to A*β* effects when stimulated at low frequencies. It is well known that the synaptic components recruited by different stimulation frequencies vary [42] and that stimulation at higher frequencies favors the recruitment of inhibitory components [42]. Inhibitory neurons and synapses seem to be more sensitive to the effects of A*β* [17, 54], which might explain why A*β* had a major effect on hippocampal input to the PFC when tested at high-frequency stimulation. In fact, this finding is consistent with the observation that fast oscillatory activity, which relies heavily on inhibitory networks [54, 55], is more sensitive to the effects of A*β* [35, 44, 45, 54] compared to slow oscillatory activity. Moreover, fast oscillatory activity is more disrupted both in AD patients [56, 57] and in AD animal models [54, 58, 59]. Thus, understanding the cellular basis of the changes in neural network activity and the alteration in neural network coupling induced by A*β* would help to explain the cellular basis of AD pathophysiology and also would reveal therapeutic strategies to reactivate such networks or reestablish their connections in order to palliate AD symptoms [60–62].
